# Notes and Notices

**Published:** 1894-01

**Authors:** 


### NOTES AND NOTICES.

SOUTHERN PINES, MOORE COUNTY, NORTH CAROLINA, is a new winter health resort just coming into prominent notice. It is located in the high dry long leaf pine sand hills amid the tar, pitch, and turpentine district. Thousands of Northern invalids have visited the place and many remarkable cures have been effected. Prominent physicians have visited the place for investigation and without a single exception say, it is the best in the United States, and we are specially requested by Mr. John T. Patrick, Commissioner of Immigration for the Southern States, to invite physicians of the Northern and Western States to visit the place and investigate in the interest of their patients.

Any physician desiring information can address Mr. Patrick at Pine Bluff, N. C.

PROFESSIONAL REPARTEE.—Apropos of the lawyers pitching into experts on the witness stand in murder trials, the case is recalled where the lawyer looked quizzically at the doctor who was testifying, and said:

"Doctors sometimes make mistakes, don't they?"

"The same as lawyers," was the reply.

"But doctors' mistakes are buried six feet under ground," said the lawyer.

"Yes," said the doctor, "and lawyers' mistakes are sometimes hung on a tree."—*Boston Herald.*

MUSICAL CONTEST.—We have received from the publishers the two great rival marches: *Protective Tariff Grand March* and *Free Trade Grand March.* The former is by the well-known author, Will L. Thompson, of East Liverpool, Ohio. The latter is by Wm. Lamartine, an author of equal talent, and both pieces are beautiful, bright, and showy marches of medium difficulty for the piano or organ. Price, 40 cents each. They are for sale at all music stores, or may be procured from Mr. Thompson at one-half price. One firm alone has ordered 15,000 copies.

